# Experimental approaches to evaluate activities of cytochromes P450 3A

**DOI:** 10.2478/v10102-010-0032-0

**Published:** 2010-11

**Authors:** Lucie Bořek-Dohalská, Petr Hodek, Jiří Hudeček, Marie Stiborová

**Affiliations:** Department of Biochemistry, Faculty of Science, Charles University, Prague, Albertov 2030, 128 40 Prague 2, CZECH REPUBLIC

**Keywords:** cytochrome P450 3A, α-naphthoflavone, metabolism

## Abstract

Cytochrome P450 (CYP) is a heme protein oxidizing various xenobiotics, as well as endogenous substrates. Understanding which CYP enzymes are involved in metabolic activation and/or detoxication of different compounds is important in the assessment of an individual's susceptibility to the toxic action of these substances. Therefore, investigation which of several *in vitro* experimental models are appropriate to mimic metabolism of xenobiotics in organisms is the major challenge for research of many laboratories. The aim of this study was to evaluate the efficiency of different in vitro systems containing individual enzymes of the mixed-function monooxygenase system to oxidize two model substrates of CYP3A enzymes, exogenous and endogenous compounds, α-naphtoflavone (α-NF) and testosterone, respectively. Several different enzymatic systems containing CYP3A enzymes were utilized in the study: (i) human hepatic microsomes rich in CYP3A4, (ii) hepatic microsomes of rabbits treated with a CYP3A6 inducer, rifampicine, (iii) microsomes of Baculovirus transfected insect cells containing recombinant human CYP3A4 and NADPH:CYP reductase with or without cytochrome b_5_ (Supersomes™), (iv) membranes isolated from of *Escherichia coli*, containing recombinant human CYP3A4 and cytochrome b_5_, and (v) purified human CYP3A4 or rabbit CYP3A6 reconstituted with NADPH:CYP reductase with or without cytochrome b_5_ in liposomes. The most efficient systems oxidizing both compounds were Supersomes™ containing human CYP3A4 and cytochrome b_5_. The results presented in this study demonstrate the suitability of the supersomal CYP3A4 systems for studies investigating oxidation of testosterone and α-NF *in vitro*.

## INTRODUCTION

The cytochrome P450s (CYP) are a family of hemoprotein enzymes that play important roles in the metabolism of drugs and carcinogens, as well as endogenous compounds such as prostaglandins, fatty acids and steroids (Gonzalez and Gelboin, 1992, Ortiz de Montellano 1995).

CYP3A is one of the major subfamilies expressed in human livers and is found at high levels in the intestinal tract (Hosea *et al*., 2000). This enzyme oxidizes endogenous and exogeneous compounds as well as over half of the drugs in therapeutic use (Hosea *et al*., 2000). The human CYP3A subfamily expressed in human livers consists of CYP3A4 (Beaune *et al*., 1986), CYP3A5 (Aoyama *et al*., 1989, Yamaori *et al*., 2003), and CYP3A7 (Kitada *et al*., 1985, Yamaori *et al*., 2003). CYP3A4 is the most abundant form of CYP3A (~30% of total CYP) expressed in adult human livers (Shimada *et al*., 1994). CYP3A enzymes are induced by rifampicin (RIF) in the human (CYP3A4/5) and rabbit (CYP3A6), but not in rat (CYP3A1/2). CYP3A4 demonstrates homotropic cooperativity (non-Michaelis-Menten kinetics) with a number of substrates (Atkins 2005, Ekins *et al*., 2003, Guengerich 1999, Tsalkova *et al*., 2007, Ueng *et al*., 1997). The enzyme is also known to exhibit heterotropic cooperativity, which is characterized by increased oxidation of one substrate in the presence of an effector, such as α-naphthoflavone (α-NF), that may also serve as a substrate or inhibitor (Galetin *et al*., 2002, Harlow and Halpert 1998, Koley *et al*., 1997, Shou *et al*., 1994, Tang and Stearns 2001, Tsalkova *et al*., 2007). During the past decade understanding of the mechanism of CYP3A4 cooperativity has progressed from a static model with multiple binding sites (Domanski *et al*., 1998, Shou *et al*., 1994, Tsalkova *et al*., 2007, Ueng *et al*., 1997) to more complex dynamic model suggesting effector-induced conformational rearrangements of the enzyme along with multiple ligand binding (Atkins *et al*., 2001, Isin and Guengerich 2006).

CYP3A4 cooperativity may be influenced by the levels of the redox partners, such as cytochrome b_5_ or cumene hydroperoxide, relative to the CYP (Kumar *et al*., 2005, Ueng *et al*., 1997). Cytochrome b_5_ has been reported to stimulate CYP3A4, dependent on the specific substrate (Kumar *et al*., 2005, Patki *et al*., 2003, Yamazaki *et al*., 1999). The mode of action of cytochrome b_5_ remains controversial (Kumar *et al*., 2005). Although the role of this protein as a source of electrons for CYPs is well known (Guryev *et al*., 2001, Schenkman and Jansson 1999, Yamazaki *et al*., 2001), increasing evidence points to an allosteric effects of cytochrome b_5_ mediated in part by an effect on the CYP spin state (Reed and Hollenberg 2003a, Reed and Hollenberg 2003b). Its modulatory effect is further supported by the fact that cytochrome b_5_ not only increases CYP activity but in some cases also inhibits its activity (Reed and Hollenberg 2003b, Yamaori *et al*., 2003). In addition, the interaction with cytochrome b_5_ may affect the degree of oligomerization of CYP in membrane (Yamada *et al*., 1995).

Understanding which CYP enzymes are involved in the metabolic activation and/or detoxication of xenobiotics and endogenous compounds is important in the assessment of an individual's susceptibility to the toxic action of these substances. Therefore, investigation which of several *in vitro* experimental models are appropriate to mimic metabolism of xenobiotics in organisms is the major challenge for research of many laboratories.

The aim of the present work was to evaluate the efficiency of different *in vitro* systems containing individual enzymes of the mixed-function monooxygenase system to oxidize two model substrates of CYP3A, exogenous (α-NF) and endogenous (testosterone) compounds, respectively.

## MATERIALS AND METHODS

### Chemicals

Glucose-6-phosphate, NADP^+^, NADPH, α-NF, 3-[(3-cholamidopropyl)dimethyl-ammonio]-1-propane sulfonate (CHAPS), dilauroyl phosphatidylcholine, and dithiothreitol were obtained from Sigma Chemical Co. (St. Louis, MO, USA). Testosterone and 6β-hydroxytestosterone were purchased from Merck (Darmstadt, Germany). Glucose-6-phosphate dehydrogenase was from Serva (Heidelberg, Germany). Bicinchoninic acid was from Pierce (Rockford, IL, USA). All chemicals were of a reagent grade or better.

### Animals and pretreatment

Adult male rabbits (2.5–3.0 kg, VELAZ, The Czech Republic) were fed *ad libitum* on pellet chow and water one week before treatment. Then, rabbits were pretreated with RIF (50 mg/kg in 40 mM NaOH i.p. for 3 consecutive days) and used for isolation of microsomes.

### Preparation of microsomes, isolation of enzymes and assays

Microsomes were isolated from livers of rabbits pretreated with RIF as described previously (Stiborova *et al*., 1995, Stiborova *et al*., 1990) and stored in 0.5 ml aliquots in liquid nitrogen until use. CYP3A6 was isolated from liver microsomes of rabbit induced by RIF. The procedure was analogous as described previously (Haugen and Coon 1976, Yang *et al*., 1985). Rabbit liver NADPH:CYP reductase was purified as described earlier (Yasukochi *et al*., 1979). Protein concentrations were assessed using the bicinchoninic acid protein assay with serum albumin as a standard (Wiechelman *et al*., 1988). Total CYP content was measured based on complex of reduced CYP with CO (Omura and Sato 1964). Supersomes™ were from Gentest corp. (Woburn, MA). Membranes isolated from of *Escherichia coli*, containing human CYP3A4 was a gift from Dr. Soucek (National Institute of Public Health, Prague, Czech Republic). Purified human CYP3A4 was from Prof. Anzenbacher (Palacky University, Olomouc, Czech Republic) and human microsomes from Dr. Szotakova (Charles University, Hradec Kralove, Czech Republic).

### Testosterone 6β-hydroxylation

The incubation mixtures for measuring the testosterone metabolism contained in a final volume of 0.5 ml: 0.1 M potassium phosphate buffer, pH 7.4, 50 µM testosterone (2 µl of stock methanol solution per incubation), 10 mM MgCl_2_, 10 mM D-glucose 6-phosphate, 1 mM NADP^+^, 1 U/ml D-glucose 6-phosphate dehydrogenase and one from the used enzyme system: (i) human hepatic microsomes (0.2 µM CYP), (ii) hepatic microsomes of rabbits treated with a CYP3A6 inducer, rifampicine (0.2 µM CYP), (iii) microsomes of Baculovirus transfected insect cells containing recombinant human CYP3A4 (0.05 µM) and NADPH:CYP reductase with or without cytochrome b_5_ (0.2 µM) (Supersomes™), (iv) membranes isolated from of *Escherichia coli*, containing recombinant human CYP3A4 (0.05 µM) and cytochrome b_5_ (0.2 µM) reconstituted with NADPH:CYP reductase (0.05 µM), and (v) purified human CYP3A4 (0.05 µM) or rabbit CYP3A6 (0.2 µM) reconstituted with NADPH:CYP reductase ( 0.05 or 0.2 µM) with or without cytochrome b_5_ (0.2 or 0.8 µM) in liposomes. Microsomes and Supersomes™ were diluted on the concentration mentioned above. Bacterial membranes were reconstituted 10 min with NADPH:CYP reductase and cytochrome b_5_ and then diluted with buffer on the used CYP concentration (see above). Reconstitution of purified CYP3A4 and CYP3A6 with NADPH:CYP reductase was carried out essentially as described earlier (Burke *et al*., 1985). Briefly, CYP3A were reconstituted as follows (0.5 µM CYP3A, 0.5 µM NADPH:CYP reductase, 0.5 µg/µl CHAPS, 0.1 µg/µl vesicles (from D,l-dilauroylphosphatidylcholine), 3 mM reduced glutathione and 50 mM HEPES/KOH, pH 7.4). An aliquot containing 25 pmol of reconstituted CYP3A4 or 100 pmol of reconstituted CYP3A6 was added to incubation mixtures. The mixtures were incubated for 15 min, at 37 °C in a shaking incubator. The reaction was terminated by addition of 0.1 ml of 1 M aqueous Na_2_CO_3_ containing 2 M NaCl. Then, phenacetin (5 µl of 1 mM stock solution) was added as an internal standard. The metabolites were extracted with 2 ml of CH_2_Cl_2_ and the extracts were evaporated to dryness. The residues were dissolved in the mobile phase for HPLC (see below).

### HPLC conditions

Testosterone and its metabolites were separated on Nucleosil (C18) HPLC column (4.6 × 25 mM, 5 µm, Macherey-Nagel, Germany). The flow rates, mobile phases and detection wavelengths for assays were 0.6 ml/min, 70:30 CH_3_OH/H_2_O (v/v), and 254 nm, respectively.

### Oxidation of α-NF

Incubation mixtures contained in a final volume of 0.5 ml: 0.1 M potassium phosphate buffer, pH 7.4, 150 µM α-NF (2 µl of stock methanol solution per incubation), 50 µl of NADPH-generating system (see above) and the same enzyme systems as in the case of testosterone oxidation (see above)**. The mixtures were incubated for 30 min at 37 °C in a shaking incubator. The reaction was terminated by addition of 0.1 ml of 1 M aqueous Na_2_CO_3_ containing 2 M NaCl. The α-NF metabolites were extracted with 2 ml of CH_2_Cl_2_ and the extracts were evaporated to dryness. The residues were dissolved in the mobile phase for HPLC. Samples were analyzed by HPLC as described elsewhere (Hosea *et al*., 2000, Thakker *et al*., 1981) to identify α-NF oxidation products. Two metabolites with retention times of 13.0 and 21.0 min (Figure 2), which were previously assigned as the trans-7,8-dihydrodiol and 5,6-epoxide were formed (Andries *et al*., 1990, Hosea *et al*., 2000, Shou *et al*., 1994). Mass spectroscopy (MALDI-TOF using α-cyano-4-hydroxycinnamic acid as a matrix) of the metabolite with a retention time of 13.0 min gave molecular ions at m/z 307 (M+H)^+^ and m/z 329 (M+Na)^+^, suggesting a dihydrodiol derivative. The metabolite with a retention time of 21.0 min gave molecular ions at m/z 289 (M+H)^+^ and m/z 311 (M+Na)^+^ and peaks at m/z 273 (M+H)^+^ and at m/z 295 (M+Na)^+^, that is an indicative of an epoxide metabolite. The results are consistent with a previous studies on the metabolism of α-NF by rat microsomes pretreated with 3-methylcholanthere (Andries *et al*., 1990) and by purified reconstituted CYP3A4 (Shou *et al*. 1994), in which these two metabolites were identified as trans-7,8-dihydrodiol (retention time = 13.0 min) and 5,6-epoxide (retention time = 21.0 min). Another minor metabolite eluted with retention time of 9.6 min has not been identified yet (Figure 2). Two peaks eluted with retention times of 26.6 and 28.2 min seem not to be the products of α-NF oxidation, because they are also present in the control reaction samples. These samples containing all reaction components were immediately (without incubation) applied on a HPLC column to analyze.

## RESULTS

Several different systems containing CYP3A enzymes were utilized to investigate oxidation of testosterone and α-NF *in vitro*: (i) human hepatic microsomes rich in CYP3A4, (ii) hepatic microsomes of rabbit treated with CYP3A6 inducer, rifampicine, (iii) microsomes of Baculovirus transfected insect cells containing recombinant human CYP3A4 and NADPH:CYP reductase with or without cytochrome b_5_ (Supersomes™), (iv) membranes isolated from of *Escherichia coli*, containing human CYP3A4 with or without cytochrome b_5_, and (v) purified human CYP3A4 or rabbit CYP3A6 reconstituted with NADPH:CYP reductase with or without cytochrome b_5_ in liposomes.

**Figure 1:**
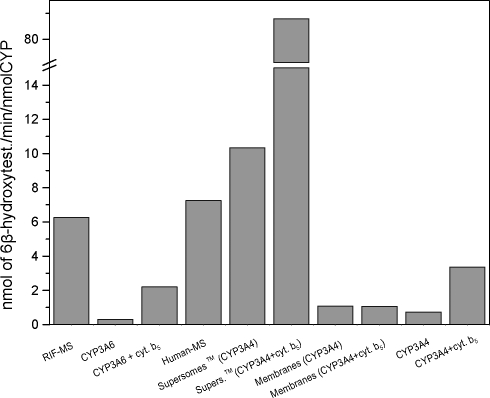
6β-Hydroxylation of testosterone by enzymatic systems containing CYP3A4 or CYP3A6.

The most efficient systems oxidizing testosterone to its 6β-hydroxylated metabolite were Supersomes™ containing human CYP3A4 and cytochrome b_5_ (Figure 1), while low efficiency of CYP3A4 and/or CYP3A6 expressed in membranes of *E. coli* or reconstituted with NADPH:CYP reductase in liposomes were found (Figure 1). The activity of purified CYP3A4 or CYP3A6 reconstituted with NADPH:CYP reductase (measured as testosterone 6β-hydroxylation) was enhanced by cytochrome b_5_. This protein did not influence oxidation of testosterone by recombinant CYP3A4 of *E.coli* membranes.

**Figure 2:**
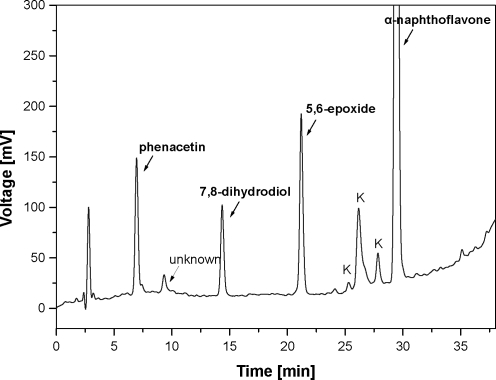
HPLC separation of α-NF metabolites by HPLC formed by incubations of 150 µM α-NF with human microsomes (100 pmol CYP) and NADPH-generating system. See Materials and Methods for details. 7,8-dihydrodiol, 5,6-epoxide and unknown metabolite were found. Peaks assigned as K are present also in control incubations without NADPH-generating system. Phenacetin was used as an internal standard.

**Figure 3:**
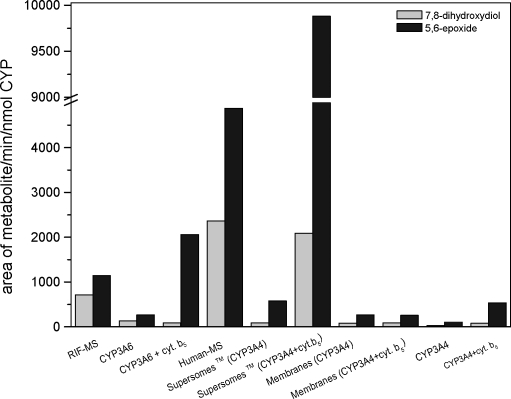
α-NF oxidation by enzymatic systems containing CYP3A4 or CYP3A6.

α-NF was oxidized into two metabolites, tentatively identified as trans-7,8-dihydrodiol (retention time = 13.0 min) and 5,6-epoxide (retention time = 21.0 min) by mass spectra and previously reported data (Andries *et al*., 1990, Shou *et al*., 1994) (Figure 2). Under the condition used, 5,6-epoxide was the major product in all systems used. The most efficient system oxidizing α-NF was Supersomes™ containing human CYP3A4 and cytochrome b_5_ (Figure 3). Also low efficiency of CYP3A4 and/or CYP3A6 expressed in membranes of *E. coli* or reconstituted with NADPH:CYP reductase in liposomes to oxidize α-NF were found (Figure 3). Their activity was enhanced by cytochrome b_5_, while cytochrome b_5_ did not influence α-NF oxidation by recombinant CYP3A4 of *E.coli* membranes**.

Surprisingly, although 6β-hydroxylation of testosterone catalyzed by recombinant human CYP3A4 and NADPH:CYP reductase without cytochrome b_5_ in Supersomes™ was very effective, α-NF oxidation was low in this system (Figures 1 and 3).

## DISCUSSION

In this report we evaluated CYP3A activity in different enzymatic systems (microsomal and/or reconstituted systems). Under the condition used, the most efficient systems oxidizing testosterone to its 6β-hydroxylated metabolite and α-NF to trans 7,8-dihydrodiol- and 5,6-epoxide were Supersomes™ containing human CYP3A4 and cytochrome b_5_. It has been shown that some CYP3A4 activities are dependent on cytochrome b_5_, specific lipid mixtures, cholate, buffer and salt composition (Shimada and Yamazaki 1998, Yamazaki *et al*., 1996).The results of this study clearly show that the effect of cytochrome b_5_ on CYP3A4-mediated oxidation of testosterone was different from that on oxidation of α-NF. This protein seems to be essential for the effective conversion of α-NF to its metabolites. Namely, 6β-hydroxylation of testosterone catalyzed by recombinant human CYP3A4 and NADPH:CYP reductase in Supersomes™ was effective even without cytochrome b_5_. On the contrary α-NF oxidation by this enzymatic system was negligible without cytochrome b_5_.

The results presented in this study demonstrate the suitability of the supersomal CYP3A4 systems for studies investigating oxidation of testosterone and α-NF *in vitro*.

The data indicate that results found in a variety of studies employing different enzymatic systems to evaluate the degree of participation of individual CYP enzymes in xenobiotic metabolism should be carefully interpreted. In order to properly evaluate a role of individual CYPs in metabolism, combination of various *in vitro* and *in vivo* approaches should be utilized.
